# Investigations into Replacing Calcined Clay with Sewage Sludge Ash in Limestone Calcined Clay Cement (LC3)

**DOI:** 10.3390/ma18040782

**Published:** 2025-02-11

**Authors:** Hui Gu, Zhaobo Meng, Yilei Wang, Xiaohui Gao, Ruihua Wang, Dongfang Wang, Jianxiong Sheng, Junjie Wang

**Affiliations:** 1 School of Architecture and Engineering, Liaocheng University, Liaocheng 252000, China; guhuiyatou@163.com (H.G.);; 2 Department of Civil Engineering, Tsinghua University, Beijing 100084, China; 3 Beijing Daxing International Airport North Line Expressway Co., Ltd., Beijing 100176, China; 4 Wastewater Treatment Plant, Jia Ming Industrial Park, Liaocheng 252000, China

**Keywords:** sludge ash, calcination temperature, LC3, compressive strengths, microstructure

## Abstract

The effects of replacing calcined clay with sewage sludge ash (SSA) treated under room temperature and high temperature ranging from 500 °C to 900 °C in limestone calcined clay cement (LC3) have been investigated in this paper. The optimal calcination temperature for SSA was found to be 800 °C based on the results of strength and microstructure observations. The main inorganic components of sludge ash are Fe_2_O_3_, SiO_2_, Al_2_O_3_, and CaO, which are very similar to the components of calcined clay in LC3, but with a very high content of Fe_2_O_3_ (55–61%) and P_2_O_5_ (9–10%). With different levels of the replacement of calcined clay with calcined SSA, setting time, compressive strength, XRD, TG/DSC, and SEM analyses of the modified LC3 pastes were conducted to identify the chemical compositions, physical properties, hydration products, microstructure, and the heavy metal contaminants within the pastes, which were compared to the results for normal LC3 paste. The incorporation of SSA significantly altered the morphologies of Ca(OH)_2_ and CaCO_3_, as well as modified the microstructure of the LC3 paste. In comparison to the pure OPC group, the LC3 pastes containing SSA exhibited a reduced Ca(OH)_2_ content and an increased CaCO_3_ content. Furthermore, the modified LC3 pastes with calcined SSA effectively facilitated the immobilization of heavy metal ions in SSA. The findings indicate the potential viability of utilizing calcined SSA as a replacement for calcined clay in LC3.

## 1. Introduction

Wastewater treatment processes inevitably produce sludge, a solid waste that presents considerable challenges owing to its elevated levels of organic material and pathogenic microorganisms. Its complex composition and substantial volume necessitate proper disposal to avoid severe environmental contamination and potential risks to human health [1,2,3]. Globally, it is estimated that sewage sludge production ranges between 70 and 105 million tons annually [4]. Similarly, China has witnessed a yearly increase of 10% in sewage sludge generation [5]. Heightened environmental awareness has led to the abandonment of traditional disposal methods, including ocean dumping and landfilling at the site of production [6].

Sludge also contains heavy metals like copper, zinc, lead, and mercury, along with toxic and non-biodegradable compounds, which strictly limit its agricultural use under current regulations. Currently, sludge incineration stands as a pivotal method of disposal, particularly in European countries where it accounts for over 50% of total sludge management [7]. Incineration effectively eliminates substantial amounts of organic matter and pathogens, dramatically reduces sludge volume, and stabilizes harmful elements within the resultant ash. Additionally, the abundant organic content in sludge can serve as an alternative fuel source for thermal power generation.

However, the management of the significant ash volumes produced during incineration poses an immediate challenge. Typically, sewage sludge ash (SSA) is landfilled or discarded directly, posing risks of secondary soil and groundwater pollution. Considering its high content of Fe_2_O_3_, SiO_2_, CaO, and Al_2_O_3_, SSA is similar to commonly used supplementary cementitious materials [8]. Consequently, SSA has found applications in various fields: in mortars [9], concrete blends [10,11,12], brick production [13], as finely granular aggregates in mortars [14], and even in asphalt paving compositions [15]. Incorporating these additional cementitious materials can notably decrease the amount of cement utilized, subsequently mitigating the carbon footprint of the cement production sector. Hence, incorporating SSA into cementitious materials has emerged as a promising trend in solid waste disposal.

To meet the demands of sustainable manufacturing and applications of Ordinary Portland Cement (OPC), in recent years, a cement called Limestone Calcined Clay Cement (LC3) has been studied. In LC3, alumina from calcined clay as well as carbonate from limestone promote synergistic interactions between the three key components, in addition to the traditional pozzolanic reaction and limestone packing reaction [16,17,18]. Now, when considering the use of Supplementary Cementitious Materials (SCMs), LC3 appears as a highly promising alternative [18].

The mineralogical profile and chemical constituents of SSA closely mirror those of silica, clay, iron oxide, and alumina [19], suggesting the feasibility of replacing the clay in LC3 with SSA. Commonly, LC3 mixtures comprise 50% clinker, 30% calcined clay, 15% limestone, and a 5% inclusion of gypsum [20,21,22,23,24,25,26]. In this study, SSA is utilized as a substitute for clay in LC3, while other curing agents are combined to create multifunctional gels. The analysis focuses on assessing the substitution strength and micromorphology of the resulting material.

The present research utilized sludge incineration ash primarily sourced from nearby waste incineration plants. The physicochemical attributes of this ash varied due to discrepancies in the incineration treatment processes employed. Additional studies are necessary to explore the effects of different calcination temperatures on SSA’s physicochemical characteristics and its pozzolanic reactivity. Naamane [27] conducted a study assessing the impact of SSA treated at temperatures ranging from 300 °C to 800 °C on cementitious materials. The findings indicated that the calcination process of sludge altered the microstructure of SSA and enhanced its pozzolanic activity, and the activity peaked at 800 °C. Furthermore, researchers found that maintaining an incineration temperature of 800 °C for a duration of 2.5 h yielded the highest compressive strength for the mortars after a period of 90 days.

Based on the above summarization, this work first investigates the influence of calcination temperature on the physical and chemical characteristics of SSA and the strength of LC3 with SSA replacing the calcined clay at different calcination temperatures to determine the optimal calcination temperature for SSA. Then, different amounts of SSA treated under the optimal calcination temperature were used to replace the clay component in LC3. The mechanical properties, hydration products, microscopic properties, and heavy metal contaminants were analyzed to finally determine the feasibility of replacing the clay in LC3 with SSA.

## 2. Materials and Methods

### 2.1. SSA Preparation

The SSA employed in this study originated from the Liaocheng Jiaming Wastewater Treatment Facility, located in Liaocheng, China. Initially, the SSA underwent pretreatment with chemical agents, including sulfuric acid, followed by dewatering through plate–frame pressure filtration. These two processes reduced the water content of the sludge to below 65%. Subsequently, the SSA used in this research was dried outdoors under sunlight and then further dried over 24 h at 105 °C. Subsequently, the sludge underwent crushing to produce smaller fragments for the calcination process.

Prior to incineration, the sludge was crushed using a mill produced by Jinbaite Electrical Appliances (FTT-1000C, Dongguan, China). This equipment, capable of handling 4500 g of material, ensured the sludge was reduced to smaller fragments suitable for the calcination process. The crushing duration for this phase was approximately fifteen minutes. Following calcination, the resulting ash underwent the same crushing process using the same type of machine.

The sludge, once crushed, was subjected to incineration at various temperatures, namely 600 °C, 700 °C, 800 °C, and 900 °C, within a high-temperature furnace, with a heating rate of 10 °C per minute. To ensure complete removal of organic matter and sufficient calcination of the sludge, the calcination time was set to 6 h. After the calcination process was concluded, the furnace, which had been operating at high temperatures, was shut down. Immediately thereafter, the calcined sludge was swiftly extracted and permitted to cool down spontaneously.

**Figure 1:**
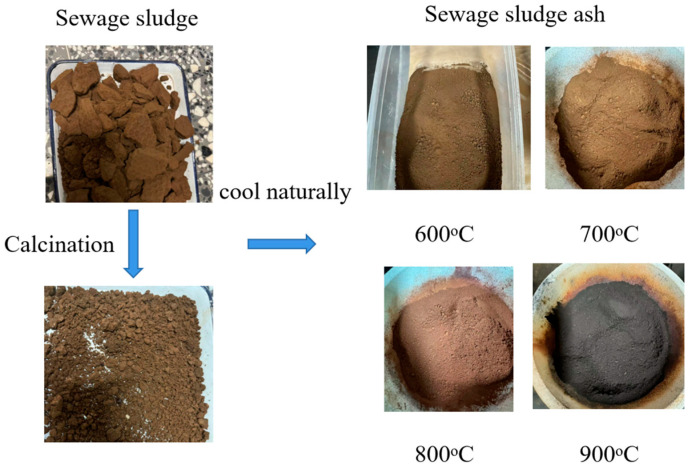
The manufacturing process of SSA.

All sludge incineration ash was then placed in a crusher, powdered twice, and sieved using a 0.6 mm sieve. Figure 1 depicts the visual characteristics and structural form of the sludge ash resulting from various calcination temperatures.

### 2.2. Raw Materials

In this investigation, the raw materials utilized included P·O 42.5 cement, SSA (derived from sludge incineration), calcined clay, fly ash, calcium oxide (CaO), limestone, and gypsum. The P·O 42.5 cement adhered to the specifications outlined in the Chinese standard GB 175-2007 [28] and corresponded to the classification CEM II/A-M 42.5N, as stipulated by BS EN197-1:2011 [29]. The composition, both mineralogical and chemical, of the diverse materials underwent analysis through X-Ray fluorescence spectroscopy (XRF), and their physical attributes are presented in Table 1. Specifically, No. S6 denotes sludge incineration ash derived from sludge calcined at 600 °C.

When comparing the oxide composition of the raw sludge to that of the sludge ash, it was observed that the calcination process, due to the decomposition of organic matter, led to an elevation in the concentration of various oxides within the ash. The sludge ash contained a significant amount of Fe_2_O_3_, attributed to the use of a wastewater phosphorus remover. Typically, the siliceous or aluminum-silica fractions within the amorphous phase are the source of the pozzolanic reactivity in supplementary cementitious materials [30]. Table 1 illustrates that as the calcination temperature rose from 600 °C to 800 °C, there was an augmentation in the amorphous phase content within the sludge ash. However, upon further elevation of temperature to 900 °C, a declining trend was observed in the amount of amorphous phase present in the sludge ash. The maximum amorphous phase in the sludge ash was achieved at a calcination temperature of 800 °C.

Table 1 also provides detailed information about the physical characteristics of the materials employed. The particle size distribution of a material is commonly represented by the characteristic value D50, which signifies the median diameter where 50% of the particles are larger. According to Table 1, the D50 of sludge ash exceeds that of the other cementitious materials. The irregular grain morphologies and open-pore structure of SSA (sludge incineration ash) significantly contribute to its remarkably high specific surface area.

**Figure 2:**
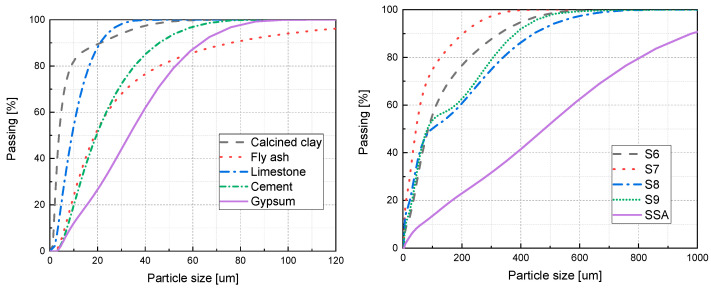
The overall distribution of particle sizes among the raw materials.

**Figure 3:**
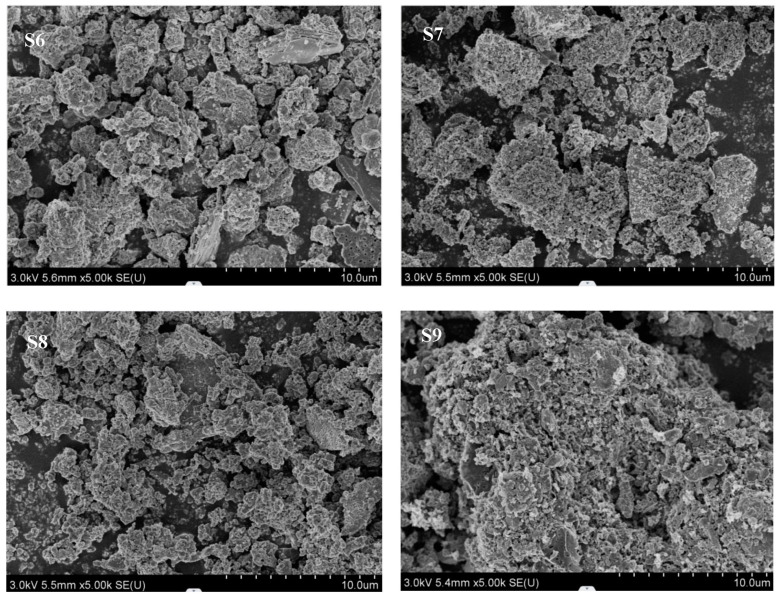
SEM images of sludge ashes (S6, S7, S8, S9) at a calcination temperatures of 600 °C, 700 °C, 800 °C, and 900 °C.

The distributions of particle sizes for all materials, as determined through laser diffraction, are depicted in Figure 2. The results indicate that SSA is considerably coarser compared to clay and fly ash (FA). Furthermore, sludge ash that has undergone incineration is finer than its pre-incineration state. Figure 3 displays the SEM (scanning electron microscope) images of sludge ash with varying calcination temperatures. It is evident from the figure that the particle sizes of S8 sludge ash are larger than those of S6, S7, and S9. The sludge ash particles exhibit irregular shapes and rough surfaces. During calcination, the breakdown of organic matter in sludge ash particles gives rise to additional residual pores, augmenting the sludge ash’s specific surface area and water uptake capacity. With increasing calcination temperature, specific components of the sludge incineration ash undergo sintering or melting processes, which modify the pore structure within the particles, potentially causing a reduction in porosity due to pore closure or alteration. Consequently, the density and particle size of the sludge incineration ash increase, while the specific surface area and water absorption rate show a decreasing trend.

### 2.3. Mixture Design

Preliminary tests were undertaken aiming to identify the optimal calcination temperature for utilizing sludge ash. In this study, Table 2 outlines the percentages of the mixtures employed. A uniform ratio of water to binder (w/b) of 0.5 was adopted across all slurry formulations. The foundational LC3 system comprised cement (50%), calcined clay (30%), and limestone (15%), by mass, with additional gypsum (5%) to sustain sulfate equilibrium. The pre-laboratory SSA30 mixtures contained 50% cement, 30% SSA subjected to various calcination temperatures between 600 °C and 900 °C, 15% limestone, and 5% gypsum. The objective of formulating diverse compositions below was to evaluate the feasibility of utilizing sludge ash as an alternative to clay in LC3. The OPC mixture consisted of 95% cement and 5% gypsum. The SSA10-FA20 mixture incorporated 50% cement, 10% SSA at the optimal calcination temperature, 20% fly ash, 15% limestone, and 5% gypsum. Meanwhile, the SSA20-CaO10 mixture comprised 50% cement, 20% SSA at the optimal calcination temperature, 10% CaO, 15% limestone, and 5% gypsum.

### 2.4. Sample Preparation and Characterizations

#### 2.4.1. Compressive Strength

Paste samples conforming to the standards set by EN 196 [31] were produced based on the binder formulations outlined in Table 2, with the objective of evaluating the development of their compressive strength. The freshly prepared pastes were cast into steel molds of dimensions 40 mm by 40 mm by 40 mm. Following this, the molds underwent mechanical vibration for a duration of one minute, were sealed using parafilm, and placed in a curing chamber set at a temperature of 20 ± 1 °C. After a 24 h curing period, the samples were demolded, enclosed in plastic bags, and placed back in the curing chamber and maintained at the same temperature. For each set, six cement paste samples were fabricated, and their compressive strengths were assessed at 3 days and 28 days of age according to the methods described in ASTM C109 [32]. The tests for compressive strength were executed using a Denison compression machine (300 kN, Jinan Shidai Gold Testing Machine Co., Ltd., Jinan, China), which was carried out according to GB/T 50081-2019 [33], and the reported values represent the mean of measurements taken from three specimens.

#### 2.4.2. The Setting Time

An assessment of cement paste setting durations based on ISO 9597:2008 [34] standards utilizing a Vicat apparatus involves specific criteria. The initial setting time is noted when the Vicat needle achieves a penetration depth of 34 ± 3 mm into the cement paste. Conversely, the final setting time is established when the needle’s penetration is limited to 0.5 mm. To validate the accuracy of both initial and final setting times, the result is an average of three measurements.

#### 2.4.3. XRD, TG/DTG and SEM

The samples intended for XRD, TG/DSC, and SEM evaluations were removed from their molds after one day and subsequently cured in a standard environment maintained at 20 ± 1 °C for a period of 28 days. To identify the hydration products in the cement pastes, XRD and TG/DTG analyses were conducted on cement paste powders. At the conclusion of the 28-day period, the cement paste samples were crushed and submerged in isopropanol to cease hydration. Subsequently, the pieces were ground into powders, dried in a vacuum oven at 40 °C for 24 h, and then subjected to XRD and TG/DTG analysis.

XRD analyses were performed using a Rigaku Smart Labs 9 kW X-ray diffractometer equipped with a kb-filtered CuKa radiation source. The scanning range was set from 5° to 65° at a rate of 5°/min. TG/DTG analyses were carried out between 20 °C and 1100 °C with a heating rate of 10 °C/min.

For scanning electron microscopy (SEM) tests, specimens were prepared from fragments of crushed cement paste. These fragments were then analyzed to observe the surface morphology of the cement paste.

#### 2.4.4. Toxicity Characteristic Leaching Procedure (TCLP)

The evaluation of the heavy metal (HM) leaching process in blended cement, conducted on samples gathered post 28 days, utilized the TCLP. Samples crushed to a particle size below 9.5 mm underwent extraction using an acetic acid solution adjusted to a pH of 2.88, with a solid-to-liquid ratio maintained at 1:2.20. The extraction containers were rotated continuously at a speed of 30 revolutions per minute (rpm) for a duration of 18 h. Following extraction, the resultant leachate was passed through a 0.45-micron filtration paper and subsequently analyzed for its minor elemental composition, encompassing silver (Ag), cadmium (Cd), chromium (Cr), copper (Cu), lead (Pb), zinc (Zn), barium (Ba), and nickel (Ni), employing Inductively Coupled Plasma–Atomic Emission Spectrometry (ICP-AES) techniques. It is worth noting that this ICP—AES method allowed us to reach detection limits of 0.0001 mg/L, which are crucial for accurately quantifying the trace amounts of heavy metals in the samples. To validate the consistency of the data, each extraction procedure was replicated three times, and the mean value derived from these repetitions was adopted. We will assess the uncertainty of the arithmetic mean and present the results in the subsequent results section to provide a more comprehensive and rigorous analysis of the data.

## 3. Results

### 3.1. The Setting Times

Recent research has documented the application of SSA as an alternative to both cement and sand within cementitious materials [9,35,36,37,38,39,40,41,42,43,44,45,46,47,48,49,50,51,52]. Generally, these investigations indicated that the incorporation of SSA decreased the setting ability of fresh mortar and prolonged the cement’s setting duration [9,37,39,42,43,44,45,46]. The table presented as Table 3 displays the setting durations of cement pastes that incorporate various materials. In the case of LC3 and SSA20-FA10 blends, a slight extension was observed in the final setting duration of the cementitious pastes. When 30% SSA was mixed into the slurry, its final setting time aligned closely with that of a standard cement slurry, whereas its initial setting time occurred sooner. For cement pastes formulated with SSA10-FA20, both the initial and final setting times were approximately similar. Conversely, cement pastes containing SSA20-CaO10 exhibited earlier setting times in both the initial and final stages. The inclusion of fly ash tended to postpone the initial setting phase. Notably, the addition of CaO significantly shortened the condensation period. However, the addition of SSA did not have much effect on the initial and final setting times of the cement paste. According to the Standard for General Silicate Cement (GB/175-2007) [28], the setting characteristics of ordinary silicate cement, when blended with mineral admixtures, should exhibit an initial setting duration exceeding 45 min, with the final setting phase lasting no longer than 600 min. From this, it can be seen that the initial and final setting time of the composite slurry mixed with sludge ash can reach the standard of general-purpose silicate cement.

### 3.2. Compressive Strengths

**Figure 4:**
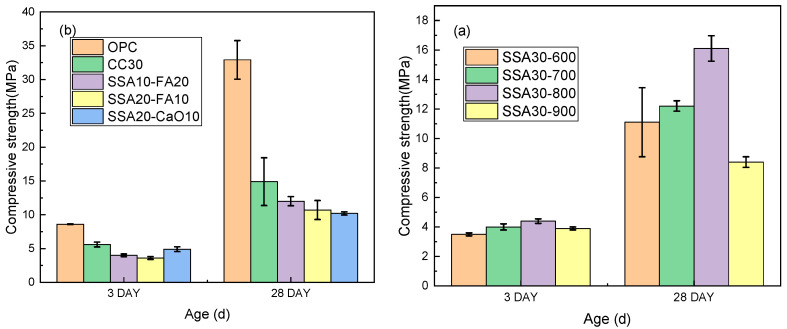
Development of the compressive strength of SSA30-600, SSA30-700, SSA30-800, SSA30-900 (**a**), OPC, LC3, SSA10-FA20, SSA20-FA10, SSA20-CaO10 (**b**) up to 3d and 28d.

Figure 4 illustrates the variations in the compressive strength of cement pastes containing varying SSA concentrations. An analysis of Figure 4a indicates that mortar mixed with sludge ash calcined at 800 °C exhibits slightly higher 3-day and significantly higher 28-day compressive strengths compared to those calcined at different temperatures. This correlation can be attributed to the smaller pore size and enhanced reactivity of S8, as depicted in Figure 3. Specifically, the 28-day compressive strength of SSA30 specimens stands at 16.1 MPa, marking a 51.1% decrease from its reference value. From Figure 4b, it becomes evident that incorporating sludge ash alongside other cementitious additives leads to a decrement in the strength of the cementitious samples. LC3 was reduced by 54.7%, SSA10-FA20 was reduced by 63.5%, SSA20-FA10 was reduced by 67.5%, and SSA20-CaO10 was reduced by 69.0%. The incorporation of supplementary cementitious materials fails to notably enhance the compressive strength of cementitious materials where sludge ash serves as a replacement. The sluggish hydration response observed in SSA–cement blends can be attributed to the retardation of the hydration process during the initial curing stage, stemming from the decreased C3S content and, concurrently, the elevated P_2_O_5_ content [27].

### 3.3. The Hydration Products

**Figure 5:**
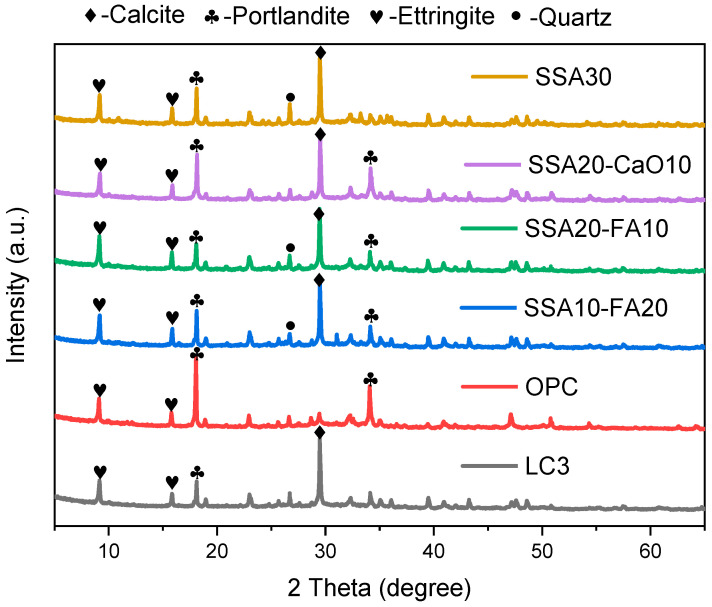
XRD patterns of cement pastes at the age of 28d.

The impact of SSA on the hydration products within cement paste can be elucidated through XRD analysis. The graphical illustrations in Figure 5 depict the XRD patterns of cement pastes formulated with varying SSA concentrations and other blends after 28 days of curing. The primary crystalline phases, i.e., calcite (CaCO_3_), portlandite (Ca(OH)_2_), ettringite (AFt), quartz (SiO_2_), are identified in these patterns. Notably, the incorporation of limestone was observed to facilitate the precipitation of calcite. SSA or clay incorporation reduces portlandite content. A factor contributing to this phenomenon is the utilization of admixtures, notably SSA, which substitute a portion of the cement, thereby resulting in a reduced cement content. Consequently, this substitution leads to a decrease in the silicate composition within the hydration products. Alternatively, another plausible explanation might lie in the reactive properties that SSA possesses, reminiscent of pozzolanic. There is a slight increase in the quartz content with the addition of SSA.

**Figure 6:**
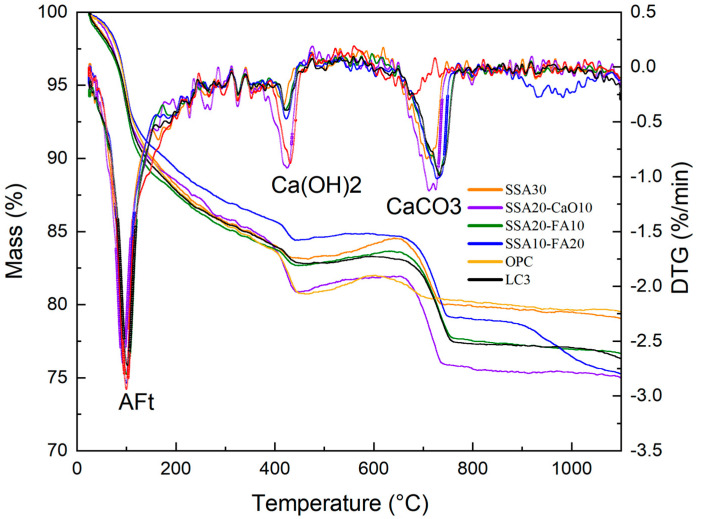
The TG/DTG curves for cement pastes at the age of 28d.

By conducting TG/DTG analyses on cement paste samples aged for 28 days, varying in SSA, fly ash (FA), and calcium oxide concentrations, a deeper understanding of SSA’s influence on the composition of calcite (CaCO_3_), portlandite (Ca(OH)_2_), ettringite (AFt), and quartz (silica) within the cement paste can be gained. Figure 6 shows the analysis results. According to the TG curves, when the temperature rose, the mass of the cement paste samples underwent a reduction. Distinct weight losses were noted at temperatures approximately 100 °C, 400 °C, and 700 °C, attributed to the decomposition of ettringite, portlandite, and calcite, respectively. The incorporation of admixtures exhibits a diluting effect, enhancing the degree of hydration of cement; consequently, polyblended cement paste demonstrates a higher hydration degree compared to pure cement paste [53,54]. With the different SSA additives, the Ca(OH)_2_ (portlandite) content becomes lower than that in pure cementitious materials. The diagram below illustrates the variation in the CaCO_3_ (calcite) contents within cement matrices that incorporate distinct SSA additives is higher than that in the ordinary cementitious materials, which is related to the addition of limestone.

### 3.4. The Microstructure Observations

**Figure 7:**
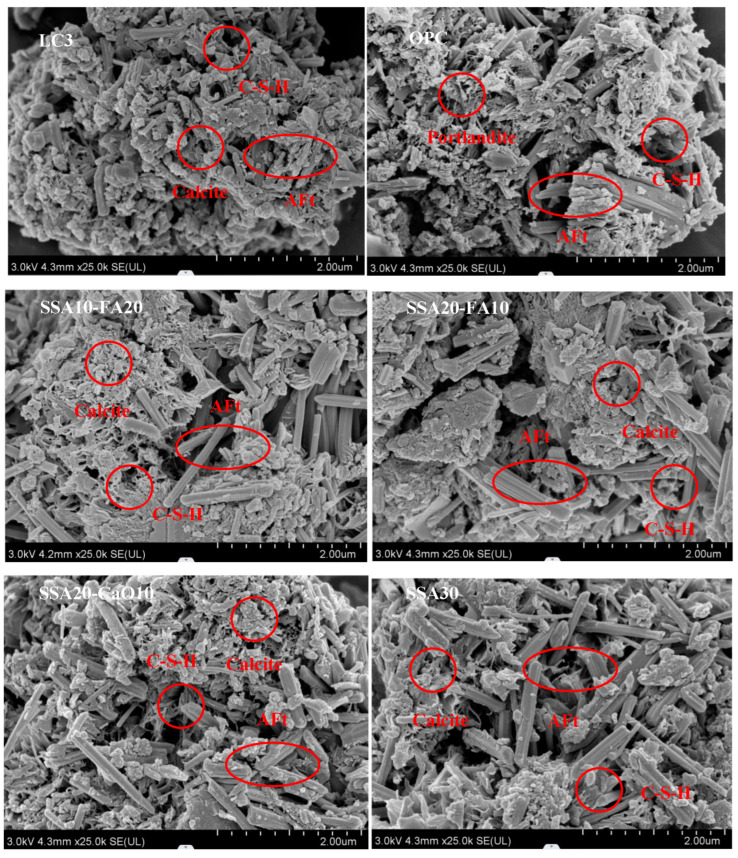
SEM images of cement pastes at the age of 28d.

The morphology of cement pastes, with varying SSA and gelling material contents, was examined using SEM. Figure 7 shows SEM images of cement pastes after 28 days with different SSA and gelling material contents. The cement paste contains low-abundance acicular calcium carbonate, contributing to a dense microstructure. In pure cement slurries, calcium hydroxide crystals are tightly packed. Upon magnification, rod-like calcite can be seen to be interspersed with the amorphous portlandite gel. In LC3 pastes with SSA, CaCO_3_ is more abundant, and Ca(OH)_2_ exhibits a radial growth pattern. SSA altered the cement paste’s morphology and increased the porosity of LC3 pastes. In samples with FA, FA interactions led to additional hydration products, filling pores and micro-fractures, forming a dense structure, and improving mechanical properties.

### 3.5. Environmental Impacts

The toxicity of cement paste extracts was determined according to the national standard of China, GB 5085.3-2007 [55], and the outcomes of this testing are presented in Table 4. For the samples tested, SSA30 leachate had the highest levels of HM, followed by SSA20-FA10. Leaching concentrations of all HMs in all samples were below the detection limits (TCLP Regulatory Limit and GB 5085.3-2007 Limit).

Currently, curing of cementitious materials and heat treatment are the two most effective methods for immobilizing heavy metal contaminants [56]. In cementitious materials, heavy metal contaminants can be embedded in the structure of the hydration product, e.g., in the laminar structure of C-S-H with needles [57]. In addition, during cement hydration, heavy metals in sludge may react with hydration products to form precipitates [58]. It was found that heavy metals can permanently bind to the formation of ettringite [59]. In addition, the dense structure of the hardened cement paste provides a low permeability barrier against leaching of heavy metals. The application of materials such as SSA in cementitious materials does not pose a threat to the environment due to the curing process of heavy metals.

## 4. Discussions

Investigations into the physicochemical characteristics of sludge ash reveal that its rough, porous surface contributes to a significant specific surface area and elevated water absorption capabilities. In comparison to OPC and standard LC3 groups, the inclusion of diverse sludge ash types was observed to augment the porosity of the mixed slurry while diminishing its strength. To gain insights into the underlying causes of these observations, we focus on two primary considerations. Firstly, the inherent porosity of sludge ash, stemming from the decomposition of organic matter during the calcination procedure (depicted in Figure 3), leads to the development of a multitude of pores within the sludge ash matrix. Secondly, the incorporation of sludge ash weakens the bonding between different hydration products, giving rise to the formation of additional pores within the slurry. However, some hydration products formed by the reaction between sludge ash, admixtures, and cement compositions can fill larger pores in the cement slurry, thereby optimizing the pore structure. Since sludge ash particles (SSA) are porous materials, and it is plausible that their pores may be partially filled by these hydration products, and their high specific surface areas provide more reaction sites.

The elevated water demand exhibited by sludge ash particles (SSA) arises due to their composition of primarily fine, sintered particles characterized by a large specific surface area. This demand for water can adversely affect the mechanical properties of mortars unless counteracted through the application of a superplasticizer. The strength of a sludge ash–fly ash–cement composite slurry is derived from both the physical filling effect and the chemical reaction effect of auxiliary cementitious materials. Consequently, the strength composition of this composite can be categorized into contributions from cement hydration, physical filling by admixtures, and chemical reactions. Mortars containing SSA typically exhibit lower compressive strengths compared to reference mortars. However, SSA exhibits a beneficial long-term impact, possibly associated with its mild pozzolanic reactivity [60]. During the initial hydration stage, excess water in the cement paste is absorbed and stored by SSA, functioning as an internal curing agent. As external humidity decreases, the water within SSA particles re-migrates and diffuses to the surrounding area, not only providing water for continued cement hydration but also preventing the relative humidity inside the cement paste from dropping too rapidly, thereby maintaining the wetting of the calcium–silicate–hydrate (C-S-H) gel [61].

The incorporation of limestone powder enhances the exothermic rate of cement hydration. By introducing fine limestone powder particles into the cement slurry, numerous nucleation sites for C-S-H gel and calcium hydroxide are created, which speeds up the crystallization, precipitation, and formation of hydration products. This, in turn, reduces the calcium ion concentration in the solution and facilitates the diffusion and migration of C3S particle surfaces into the solution, thereby accelerating the cement hydration process [62]. Reactive alumina present in clay and fly ash fosters the formation of calcite within the cement paste. Additionally, calcium carbonate transforms a portion of monosulfur hydrated calcium thioaluminate into calcite. Compared to C-S-H gels, calcite boasts a larger specific surface area and a unique lattice structure, generating higher crystallization pressure upon water absorption and causing volume expansion due to colloidal swelling upon hydration [63,64]. The production of calcium hydroxide occurs as a result of the interaction between calcium oxide and water, which increases the solid phase volume by 97% during the initial hydration stages and decreases the overall porosity of the composite system [65].

The hydration properties of LC3 slurry during the initial stages can be improved by incorporating gypsum. The inclusion of gypsum offsets the consumption of gypsum by active Al_2_O_3_ in the sludge ash, thereby enhancing the hydration process within the composite slurry. This results in an increased content of hydration products, a higher exothermic heat during cement hydration, and accelerated development of early strength in LC3.

## 5. Conclusions

This study explored the influence of calcination temperature on the physicochemical characteristics and chemical constituents of normal and calcined sewage sludge ash (SSA) as a substitute for calcined clay in LC3. The hydration products were identified and the microstructure was observed in LC3 pastes with different SSA contents replacing the clay. Based on the outcomes of the experiments conducted, the following insights and conclusions can be drawn:

(1) The introduction of SSA into modified LC3 pastes led to a slight prolongation of the final setting time. However, with 30% SSA incorporation, the final setting time of LC3 pastes approximated that of ordinary cement slurry, while exhibiting an earlier initial setting time. Replacing calcined clay with calcined SSA led to a reduction in the compressive strength of LC3 samples.
(2) As the calcination temperature rose, the amorphous phase content within the calcined SSA increased, peaking at an optimal calcination temperature of 800 °C. The primary chemical compositions of SSA, namely Fe_2_O_3_, SiO_2_, Al_2_O_3_, and CaO, which mirror the chemical compositions of the clay utilized in LC3, albeit with a higher Fe_2_O_3_ and P_2_O_5_ content in SSA.
(3) The incorporation of SSA into LC3 did not produce notable alterations in the phase compositions of the modified pastes compared to normal LC3. The primary crystalline minerals observed in the modified LC3 pastes include calcite (CaCO_3_), portlandite (Ca(OH)_2_), ettringite (AFt), and quartz (SiO_2_). Notably, compared to the pure OPC group, the Ca(OH)_2_ content was lower, while the calcite content was higher in LC3 pastes with SSA.
(4) In samples containing SSA, an increased content of CaCO_3_ was observed, and the Ca(OH)_2_ exhibited a radial morphology. The addition of SSA altered the morphologies of Ca(OH)_2_ and CaCO_3_, and modified the microstructure of the LC3 paste. Furthermore, LC3 pastes with SSA appeared to possess a more porous microstructure than the normal LC3 group.
(5) Heavy metal ions in SSA can be mostly immobilized in the LC3 pastes containing calcined SSA, thereby alleviating any possible environmental risks associated with utilizing SSA as a calcined clay substitute in LC3.

## Figures and Tables

**Table 1:** The raw materials’ constituent chemicals and inherent physical characteristics.

Material	CC	FA	LS	Cement	Gypsum	S6	S7	S8	S9	SS
Chemical composition (%)										
SiO_2_	45.54	49.89	0.10	25.48	0.37	12.53	13.96	16.06	15.82	14.67
CaO	0.10	3.53	98.03	54.45	37.09	4.57	4.12	3.61	3.69	3.23
Al_2_O_3_	51.18	36.01	0.05	7.89	0.10	4.16	4.39	4.98	5.23	4.05
Fe_2_O_3_	0.42	4.19	0.04	4.35	0.03	61.50	59.41	54.83	55.62	58.31
MgO	0.12	0.94	0.71	1.40	0.57	1.80	1.28	1.23	1.20	1.17
Na_2_O	0.70	0.69	0.59	0.60	0.52	1.17	1.27	1.41	1.35	1.71
K_2_O	0.10	1.71	--	0.64	0.02	0.81	0.92	1.08	1.06	0.97
P_2_O_5_	0.40	0.38	0.08	0.15	0.07	8.98	10.02	11.97	11.08	9.04
SO_3_	0.19	1.13	0.26	4.12	61.06	3.02	2.70	2.23	2.62	3.10
Physical property										
D_50_ (μm)	3.80	18.8	9.24	19.5	33.2	85.6	44.3	97.7	83.0	55.11
Specific surface area (m^2^/kg)	1776	420.5	837.3	393.1	283.6	184.4	349.7	303.8	213.3	479

Note: CC is calcined clay, FA is fly ash, LS is limestone, and SS is sewage sludge. Sludge ashes (S6, S7, S8, S9) were calcined at 600 °C, 700 °C, 800 °C, and 900 °C, respectively.

**Table 2:** Detailed mixture ratios for OPC and SSA-LC3 systems (wt.%).

System		w/b	OPC	CC	SSA		FA	CaO	LS	Gypsum
					Proportions	Calcination Temperature				
pre-laboratory	SSA30-600	0.5	50	0	30	600	0	0	15	5
	SSA30-700	0.5	50	0	30	700	0	0	15	5
	SSA30-800	0.5	50	0	30	800	0	0	15	5
	SSA30-900	0.5	50	0	30	900	0	0	15	5
OPC		0.5	95	0	0	0	0	0	0	5
LC3		0.5	50	30	0	0	0	0	15	5
SSA10-FA20		0.5	50	0	10	800	20	0	15	5
SSA20-FA10		0.5	50	0	20	800	10	0	15	5
SSA20-CaO10		0.5	50	0	20	800	0	10	15	5

Note: SSA30-600 means LC3 with 50% OPC, 30% SSA calcined at 600 °C, 15% limestone and 5% gypsum. SSA10-FA20 means LC3 with 50% cement, 10% SSA calcined at 800 °C, 20% fly ash, 15% limestone, and 5% gypsum.

**Table 3:** Setting times of cement pastes.

System	Initial Setting Time (min)	Final Setting Time (min)
OPC	201	298
LC3	187	492
SSA30	120	283
SSA10-FA20	245	295
SSA20-FA10	200	370
SSA20-CaO10	28	150

**Table 4:** The cement pastes exhibited specific elution levels for certain toxic elements.

Application	Concentration of HM (mg/L)							
	Ag	Cd	Cr	Cu	Pb	Zn	Ba	Ni
OPC	ND	0.0150	0.5576	0.4345	0.3952	0.8894	0.7153	ND
SSA30	ND	0.0500	1.3856	1.6498	0.9394	27.6620	1.4744	0.2084
SSA10-FA20	ND	0.0234	0.5529	0.7450	0.6131	7.3584	1.9619	0.0566
SSA20-FA10	ND	0.0383	0.9468	1.2535	0.7756	18.4746	1.0816	0.1657
SSA20-CaO10	ND	0.0323	0.9819	1.0713	0.6113	16.5055	0.8852	0.1156
TCLP ^a^ Regulatory Limit	5	1	5	15	5	25	100	25
GB 5085.3-2007 ^b^ Limit	5	1	15	100	5	100	100	5

ND: not detected; ^a^ TCLP: Toxicity Characteristic Leaching Procedure; ^b^ Chinese Standard: Identification Standards for Hazardous Wastes–Identification of extraction toxicity.

## Data Availability

The raw data supporting the conclusions of this article will be made available by the authors on request.
